# Iron Concentration Does Not Differ in Blood but Tends to Decrease in Cerebrospinal Fluid in Parkinson’s Disease

**DOI:** 10.3389/fnins.2019.00939

**Published:** 2019-09-26

**Authors:** Xiaoli Shen, Huazhen Yang, Dongfeng Zhang, Hong Jiang

**Affiliations:** ^1^Department of Physiology, Shandong Provincial Key Laboratory of Pathogenesis and Prevention of Neurological Disorders and State Key Disciplines: Physiology, Qingdao University, Qingdao, China; ^2^Department of Epidemiology and Health Statistics, Qingdao University, Qingdao, China; ^3^Department of Epidemiology and Health Statistics, West China School of Public Health, Sichuan University, Chengdu, China

**Keywords:** iron, Parkinson’s disease, blood, cerebrospinal fluid, meta-analysis

## Abstract

**Background:**

Iron accumulation in the substantia nigra in PD patients was acknowledged, but the studies on alteration of iron levels in blood and cerebrospinal fluids (CSF) reported inconsistent results.

**Objective:**

To determinate the alterations of blood and CSF levels of iron in PD patients, a case-control study and a meta-analysis both in blood and CSF were conducted.

**Methods:**

In the case-control study, 43 PD patients and 33 controls were recruited to test iron metabolism, 15 normal and 12 PD patients donated CSF. Levels in iron were quantified by inductively coupled atomic emission spectrometry. Iron metabolism was analyzed by routine blood tests. In the meta-analysis, a comprehensive literature search was performed on relevant studies published from Jan 1980 to Dec 2018 in PubMed, Web of Science and EMBASE databases. The pooled standard mean difference (SMD) with random effects model was selected to estimate the association between iron levels and PD.

**Results:**

In the case-control study, the iron level in serum in the controls and PD patients were 110.00 ± 48.75 μg/dl and 107.21 ± 34.25 μg/dl, respectively, no significant difference was found between them (*p* = 0.850), with a small effect size (Cohen’s d: 0.12; 95% CI: 0.08–0.17). Ferritin level in PD patients was lower than controls (*p* = 0.014). The CSF levels of iron in control and the PD patients were 20.14 ± 3.35 ng/dl and 16.26 ± 4.82 ng/dl, respectively. CSF levels of iron were lower in PD compared with that of controls (*p* = 0.021), with a moderate effect size (Cohen’s d: 0.51; 95% CI: 0.43–0.65). In the meta-analysis, 22 eligible studies and a total of 3607 participants were identified. Blood levels of iron did not differ significant between PD patients and the controls [SMD (95% CI): −0.03 (−0.30, 0.24)], but CSF iron levels tended to be lower in PD patients compared with that in the controls [SMD (95% CI): −0.33 (−0.65, −0.00)].

**Conclusion:**

Iron homeostasis may be disturbed in CSF, but not in the peripheral blood in PD.

## Introduction

Parkinson’s disease (PD) is the second leading neurodegenerative disease worldwide. It is caused by a progressive loss of neurons in the substantia nigra, and characterized by clinical features including resting tremor, bradykinesia, rigidity, postural instability and gastrointestinal dysfunction (Farlow et al., 1993; Lill and Klein, 2017). Although several genetic and environmental factors have been implicated (Gorell et al., 2004; Dick et al., 2007), the etiology of PD is not fully understood.

The imbalance of metals homeostasis in PD has been debated for decades. Iron was more implicated in PD for its property that is easy to generate free radical (Lee and Andersen, 2010; Sian-Hulsmann et al., 2011; Hagemeier et al., 2012; Mochizuki and Yasuda, 2012; Ayton and Lei, 2014). Iron selectively accumulation in the substantia nigra, which is acknowledged in PD (Drayer et al., 1986; Sofic et al., 1988, 1991; Dexter et al., 1989, 1992; Good et al., 1992; Wang et al., 2013), accelerates nigrostriatal neurodegeneration through oxidative stress which was generated by Fenton reaction (Sian-Hulsmann et al., 2011; Mochizuki and Yasuda, 2012) and Fe^3+^-melanin complexes formation (Youdim and Riederer, 1993; Gerlach et al., 1994). Iron elevation is also reported in familial PD, indicating a link between iron accumulation in the substantia nigra and the PD-associated proteins, such as alpha-synuclein, LRRK2, PINK1, Parkin, and DJ-1 (Hagenah et al., 2007, 2008; Schweitzer et al., 2007; Bruggemann et al., 2011). Increasing magnetic susceptibility (a surrogate marker of brain iron) in the globus pallidus and red nuclei was associated with decreasing manual dexterity (Li et al., 2015). Unlike changes in the substantia nigra, iron levels were not changed in the cerebellum, caudate nucleus, putamen, and cerebral cortex in postmortem PD brains, as well as reduced in the globus pallidus compared with control values (Dexter et al., 1989). In addition, in our previous work, iron levels were found decreased in the temporal cortex in PD postmortem brains (Yu et al., 2013).

Although the imbalance of cerebral iron metabolism in PD patients is well-documented, the change of iron levels in the peripheral blood and the cerebrospinal fluid (CSF) in PD patients was not fully identified. To date, studies on the alteration of iron levels in blood (Cabrera-Valdivia et al., 1994; Logroscino et al., 1997; Jimenez-Jimenez et al., 1998; Forte et al., 2004; Hegde et al., 2004; Gellein et al., 2008; Ahmed and Santosh, 2010; Fukushima et al., 2010, 2013; Madenci et al., 2012; Zhao et al., 2013; Kumudini et al., 2014) and CSF (Gazzaniga et al., 1992; Logroscino et al., 1997; Jimenez-Jimenez et al., 1998; Forte et al., 2004; Bocca et al., 2006; Alimonti et al., 2007a; Hozumi et al., 2011) in PD patients reported controversial results. Here, a case-control study plus a quantitative meta-analysis were performed to evaluate the alteration of iron levels in the serum and CSF in PD patients.

## Materials and Methods

### Case-Control Study

#### Clinical Samples

The study protocol was approved by the Institutional Ethics Committee of Medical College of Qingdao University (2016N012), and conducted in accordance with the Declaration of Helsinki. All patients gave written informed consent to participate. Fifty patients with PD (25 female and 25 male) over 40 years were recruited from the affiliated hospital of Qingdao university (Qingdao, Shandong, China) between Jan 2018 and Oct 2018. The PD patients were diagnosed according to the “UK Parkinson’s Disease Society Brain Bank Criteria” (Pichler et al., 2013; Zhao et al., 2013). The following exclusion criteria were applied to PD patients: (a) Ethanol intake higher than 80 g/day in the last 8 months. (b) Acute infectious disorders, traumatisms or surgery in the last 6 months. (c) Atypical dietary habits. (d) Blood donation histories. (e) Systemic diseases, including anemia, hepatosis, heart failure, pulmonary disorders and chronic renal failure. (f) Female patients who had not been through menopause. (g) PD patients with history of restless leg syndrome (RLS), periodic limb movement disorder (PLMD) and excessive daytime sleepiness (EDS).

For control purpose, 50 healthy persons (25 female and 25 male) over 40 years were included from the physical examination center of the same hospital. All these controls were affirmed with no signs of neurodegenerative disease and without proven organic disease, iron-deficiency anemia, and neurologic or psychiatric disease. After excluding the hemolytic samples, 43 patients with PD (21 female and 22 male) and 33 healthy control (19 female and 14 male) was included. Among them, 15 normal and 12 PD patients donated CSF.

#### Collection and Measurement of Sample

Blood samples were collected by venipuncture in the morning between 7 and 8 A.M after a night fasting, then centrifuged for serum and rapidly stored at −80°C for further analysis. CSF was collected from patients and controls in the acid-wash tube by lumbar puncture. Prior to analysis, 20 μl serum or CSF were added with 1 mL concentrated HNO_3_ (Yingdong, Jinan, China), and digested in the oven at 55°C overnight. After digestion, samples were diluted (1:50) with ultra pure water to detect the final iron concentration. Iron concentration was measured by inductively coupled plasma atomic emission spectrometry (Agilent Technologies 770 series), which belongs to the Qingdao water quality monitoring center. Argon gas flow rate was maintained at 16 L/min and 1 KW power was used. The standards of iron of 10, 20, 50,100, 200 μg/L were prepared and calibrated. The wavelength of detection was 259.94 nm. Quality control of the analyses was performed by analyzing certified standard reference material. Ferritins, total iron binding capacity (TIBC), transferrin saturation were analyzed in the affiliated hospital of Qingdao university using a KX21 automatic blood cell analyzer (Sysmex, Japan) for routine blood tests.

#### Statistic Analyses

Statistical analyses were conducted with Stata 15.0 software (Stata Corp., College Station, TX, United States). Means for iron levels, ferritin levels, total iron binding capacity, transferring saturation were compared using two-sample t-test. A p-value less than 0.05 were considered significant in all statistical analyses. As a standardized measure of effect size, we estimated the difference between two group using Cohen’s d. All participants in the case-control study were also included in the meta-analysis.

### Meta-Analysis

A systematic literature search from January 1980 to December 2018 was conducted using the PubMed, Web of Science, EMBASE database. The search terms were “Parkinson’s disease” OR “Parkinson” OR “PD” combined with “iron” OR “Fe.” The reference lists of retrieved articles were also reviewed to identify additional studies not captured by our database search. These articles are managed by Endnote X7 software.

For inclusion, studies had to meet the following criteria: (1) observational studies in humans, (2) original study reported the iron levels in serum, plasma or CSF, (3) sample size and iron levels (Mean ± standard error of mean or Mean ± standard deviations) were provided; (4) Iron levels both of PD patients and healthy control were given. The following exclusion criteria were also used: (1) reviews, editorials, letters without original data mentioned, (2) articles about familial PD, (3) unit of iron levels was not given.

The following data were extracted from each study by two investigators separately: the first author’s name, publish year, country in which the study was conducted, sample size, mean age of patients and controls, male’s percentage, tested method of iron, diagnostic criteria of PD, mean ± SD levels of iron in serum, plasma or CSF, fasting status, match of potential confounders. If the standard error of mean (SEM) was provided in this study, SEM was converted to standard deviation (SD) by the following formula: $\mathrm{SD}=\mathrm{SEM}\times \sqrt{n}$.

The standardized mean difference (SMD) with 95% confidence interval (CI) was calculated by using the random effects model to assess the pooled difference on iron levels between PD patients and controls. Heterogeneity among studies was assessed by I^2^ statistical measure (Higgins et al., 2003). Meta-regression with restricted maximum likelihood estimation was performed to describe the potentially important covariates. In the cumulative meta-analysis, studies were added one at a time in the order of year published, and the results were summarized sequentially. Influence analysis was conducted to investigate whether an individual study affected the pooled result or not. Small-study effect was assessed with funnel plot and Egger test. Sensitivity analysis was conducted by only including studies that excluded subjects with hepatopathy, nephropathy, atypical dietary habits, intake of iron supplements and chelating agents, as well as studies that excluded subjects with blood transfusions, blood donation anemia. All statistical analyses were performed with Stata version 15.0 software (Stata Corporation, College Station, TX, United States).

## Results

### Case-Control Study

Patients with PD and the controls did not differ for age [PD, 69.5 ± 5.9; controls, 67.6 ± 4.3; *p* = 0.26], as well as for sex distribution (χ^2^ = 0.40, *p* = 0.36). As shown in Table 1, PD patients had significantly lower ferritin level than controls (*p* = 0.014). Although serum iron, TIBC, and transferrin saturation showed a downward trend in PD patients compared with the controls, these changes did not reach statistical significance. Fifteen control (male/female, 7/8; age, 62.5 ± 4.9) and 12 PD patients (male/female, 5/7; age, 65.3 ± 5.7) donated CSF. CSF levels of iron were lower in PD patients compared with that of controls (*p* = 0.021), with a moderate effect size (Cohen’s d: 0.51; 95% CI: 0.43–0.65).

**TABLE 1:** The level of iron in CSF and iron storage in serum among control and PD groups.

	**Control (Mean ± SD)**	**PD patients (Mean ± SD)**	***P***	**Cohen’s d**	**95% CI of Cohen’s d**
Serum iron (μg/dL)	110.00 ± 48.75 (*n* = 43)	107.21 ± 34.25 (*n* = 33)	0.780	0.12	0.08∼0.17
Ferritin (ng/mL)	220.81 ± 79.46 (*n* = 43)	176.09 ± 72.42 (*n* = 33)	0.014	0.76	0.62∼0.87
Total iron binding capacity (μg/dL)	340.51 ± 30.25 (*n* = 43)	336.34 ± 24.36 (*n* = 33)	0.519	0.10	0.06∼0.12
Transferrin saturation	32.35% (*n* = 43)	31.84% (*n* = 33)	0.210	0.08	0.04∼0.11
CSF iron (ng/dL)	20.14 ± 3.35 (*n* = 15)	16.26 ± 4.82 (*n* = 12)	0.021	0.51	0.43∼0.65

### Meta-Analysis

**FIGURE 1:**
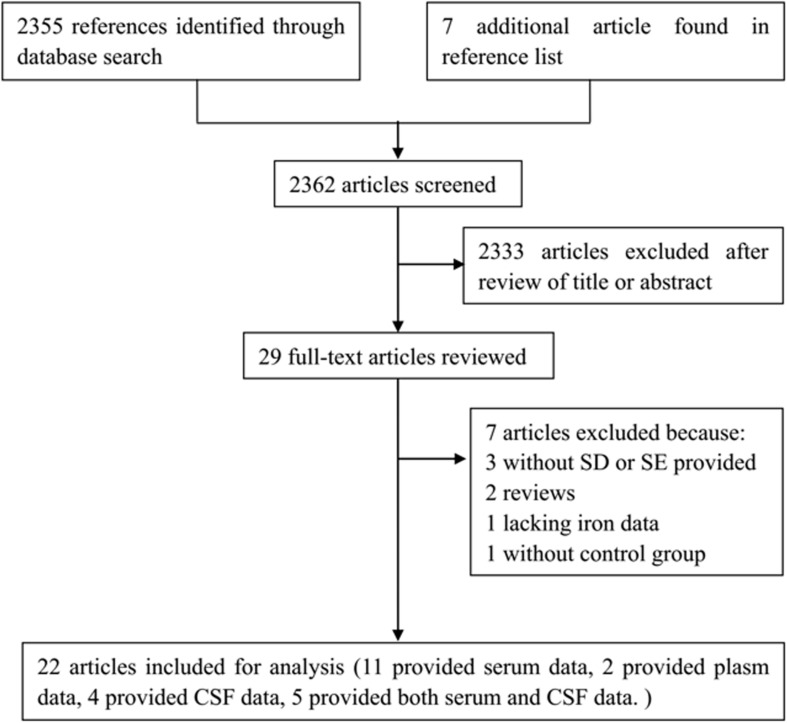
Flow of the literature.

2362 articles were identified by our literature search. 2333 articles were excluded after review of titles or abstracts. 7 studies were excluded after reviewing the full texts of 29 possibly relevant articles (Torsdottir et al., 1999; Alimonti et al., 2007b; Nischwitz et al., 2008; Speziali and Di Casa, 2009; Squitti et al., 2009; Farhoudi et al., 2012; Pichler et al., 2013). Of them, 3 articles did not provide SD or SEM (Torsdottir et al., 1999; Alimonti et al., 2007b; Squitti et al., 2009), 2 were reviews (Torsdottir et al., 1999; Speziali and Di Casa, 2009), 1 article was lacking data of iron level (Pichler et al., 2013), 1 article was lacking of control group (Nischwitz et al., 2008). For the remaining 22 articles, 13 of them reported the serum or plasma iron (Cabrera-Valdivia et al., 1994; Logroscino et al., 1997; Hegde et al., 2004; Forte et al., 2005; Gellein et al., 2008; Ahmed and Santosh, 2010; Fukushima et al., 2010, 2013; Farhoudi et al., 2012; Madenci et al., 2012; Zhao et al., 2013; Kumudini et al., 2014; Mariani et al., 2016), 4 of them reported the CSF iron (Gazzaniga et al., 1992; Bocca et al., 2006; Alimonti et al., 2007a; Hozumi et al., 2011), and 5 of them reported both serum and CSF iron (Jimenez-Jimenez et al., 1998; Forte et al., 2004; Qureshi et al., 2006; Hu et al., 2015; Sanyal et al., 2016) (Table 2). The steps of the literature search and retrieve are shown in Figure 1.

**TABLE 2:** Characteristics of included studies in the meta-analysis for iron concentration in blood and CSF.

**Study**	**Country**	**Unit**	**Parkinson’s disease**				**Controls**				**Methods to determine iron levels**	**Food fasting**
			***n***	**Sex (% male)**	**Mean Age (years)**	**Iron level (mean ± SD)**	***n***	**Sex (% male)**	**Mean Age (years)**	**Iron level (mean ± SD)**		
**Studies on serum**												
Cabrera-Valdivia et al., 1994	Spain	μg/dL	68	47.06	65.8 ± 0.96	82.7 ± 37.33	68	52.94	65.80 ± 1.00	70.9 ± 29.89	–	yes
Logroscino et al., 1997	America	μg/dL	104	–	–	28.3 ± 11.6	352	–	–	33.9 ± 15.2	AAGFM	–
Jimenez-Jimenez et al., 1998	Spain	mg/L	37	37.84	65.70 ± 8.80	1.01 ± 0.33	37	43.24	62.40 ± 17.8	0.95 ± 0.3	AAS	yes
Forte et al., 2004	Italy	μg/L	26	92.31	64.90 ± 10.80	23.54 ± 8.59	13	46.15	63.80 ± 13.70	20.29 ± 7.02	ICP-AES	yes
Forte et al., 2005	Italy	ng/mL	71	74.65	65.50 ± 9.40	1122 ± 432	44	75.00	51.90 ± 4.00	1596 ± 442	ICP-AES	–
Hegde et al., 2004	India	μol/mL	27	51.85	57.15 ± 5.20	0.02 ± 0.004	25	52.00	55.40 ± 6.40	0.023 ± 0.009	ICP-AES	–
Qureshi et al., 2006	Sweden	mg/L	19	32.00	72.00 ± 17.00	1.09 ± 0.45	21	38.09	62.00 ± 11.00	1.16 ± 0.23	AAS	yes
Gellein et al., 2008	Norway	μg/L	38	48.48	–	1251 ± 551	99	48.48	–	1146 ± 463	ICP-MS	–
Ahmed and Santosh, 2010	India	μg/dL	45	57.78	57.62 ± 9.10	110.4 ± 0.6	42	59.52	55.62 ± 3.25	123 ± 8	ICP-AES	–
Fukushima et al., 2010	China	μg/mL	58	62.00	64.20 ± 9.40	2 ± 0.83	82	57.31	63.65 ± 9.35	1.5 ± 0.78	ICP-AES	yes
Madenci et al., 2012	Turkey	μg/dL	60	55.00	68.50 ± 8.30	74.6 ± 29.29	42	52.38	66.90 ± 8.30	74.8 ± 27.11	–	yes
Farhoudi et al., 2012	Iran	mg/dL	50	56.00	64.53 ± 10.18	70.22 ± 25.18	50	50.00	63.53 ± 9.78	67.62 ± 39.53	Biochemical methods	no
Fukushima et al., 2013	China	μg/mL	58	62.00	64.30 ± 9.40	2.1 ± 0.84	81	58.02	63.70 ± 9.40	1.51 ± 0.78	ICP-AES	yes
Hu et al., 2015	China	nmol/mL	102	46.09	56.31 ± 13.38	4.12 ± 1.06	31	–	–	4.19 ± 1.05	ELISA	yes
Mariani et al., 2016	Italy	ug/dL	92	67.40	70(38–83)	79 ± 34	112	35.80	62(31–87)	86.1 ± 34.9	American Monitor KDA analyzer	yes
Sanyal et al., 2016	India	μg/L	250	64.80	57.88 ± 12.06	1156 ± 264.94	280	65.35	56.42 ± 9.68	1205.49 ± 316.09	AAS	no
**Studies on plasm**												
Zhao et al., 2013	China	μg/L	238	50.80	67.10 ± 11.30	1656 ± 749	153	50.66	67.00 ± 12.10	1470 ± 648	AAS	yes
Kumudini et al., 2014	India	ng/mL	150	71.33	55.70 ± 10.60	554.4 ± 123.8	170	70.58	53.73 ± 10.90	421.7 ± 126.1	ICP-AES	–
**Studies on CSF**												
Gazzaniga et al., 1992	Italy	μg/L	11	90.91	64.9(49–78)	181 ± 75.1	22	90.90	–	275.9 ± 153.6	AAS	–
Jimenez-Jimenez et al., 1998	Spain	mg/L	37	37.84	65.70 ± 8.80	0.17 ± 0.17	37	43.24	62.40 ± 17.80	0.21 ± 0.15	AAS	yes
Forte et al., 2004	Italy	μg/L	26	92.31	64.90 ± 10.80	33 ± 29.4	13	46.15	63.80 ± 13.70	73.3 ± 72.7	ICP-AES	yes
Qureshi et al., 2006	Sweden	μg/L	19	68.42	72.00 ± 17.00	397 ± 217.94	21	38.09	62.00 ± 11.00	237 ± 169.56	AAS	yes
Bocca et al., 2006	Italy	ng/mL	91	70.33	65.50 ± 9.70	28.2 ± 14.6	18	55.56	63.30 ± 13.80	45 ± 30.2	ICP-AES	yes
Alimonti et al., 2007a	Italy	μg/L	42	85.71	64.50 ± 10.70	28.2 ± 14.6	20	85.00	66.20 ± 14.70	35.5 ± 5.03	ICP-AES	yes
Hozumi et al., 2011	Japan	μg/L	20	45.00	68.7 ± 5.8	263.9 ± 112.9	15	40.00	48.2 ± 22.2	238 ± 54.7	ICP-MS	–
Hu et al., 2015	China	nmol/mL	51	45.10	61.23 ± 13.18	0.458 ± 0.197	31	–	–	0.495 ± 0.173	ELISA	yes
Sanyal et al., 2016	China	μg/L	50	70.00	58.72 ± 12.83	182.88 ± 89.28	60	70.00	60.11 ± 10.44	212.46 ± 33.58	AAS	no

*AAS, Atomic absorption spectrophotometer; AAGFM, Atomic absorption graphite furnace micromethod; ELISA, Enzyme linked immunosorbent assay; ICP-AES, Inductively coupled plasma atomic emission spectrometry; ICP-MS, Inductive coupled plasma mass spectroscopy.*

#### Iron Concentration Does Not Differ in Blood in Parkinson’s Disease

**FIGURE 2:**
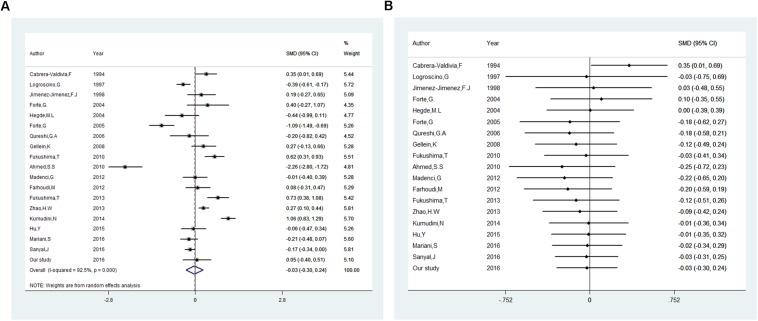
Association between blood levels of iron and Parkinson’s disease. **(A)** Forest plot of the Parkinson’s disease risk for blood iron level. **(B)** Cumulative meta-analysis of the Parkinson’s disease risk for blood iron level. The standardized mean difference (SMD) with 95% confidence interval (CI) was calculated by using the random effects model.

Besides our study, 18 studies on serum or plasma level of iron were included in this meta-analysis (16 in serum, 2 in plasma). Heterogeneous results were observed in these studies: 3 studies reported an increase of iron levels in PD patients compared with that in controls (Fukushima et al., 2010, 2013; Kumudini et al., 2014), 6 studies reported a decrease (Logroscino et al., 1997; Hegde et al., 2004; Forte et al., 2005; Zhao et al., 2013; Mariani et al., 2016; Sanyal et al., 2016), and 9 reported no difference (Cabrera-Valdivia et al., 1994; Jimenez-Jimenez et al., 1998; Forte et al., 2004; Qureshi et al., 2006; Gellein et al., 2008; Ahmed and Santosh, 2010; Farhoudi et al., 2012; Madenci et al., 2012; Hu et al., 2015). Including the subjects recruited in our study, 1555 PD cases and 1889 controls was involved. As shown in Figure 2A, no significant difference was found in serum or plasma levels of iron between PD patients and controls in this meta-analysis. The pooled SMD from random-effects model was −0.03 (95% CI: −0.30, 0.24; *I*^2^ = 92.5%). High heterogeneity (*I*^2^ = 92.5%, *P* < 0.001) was found in the analysis.

In order to explore the source of the between-study heterogeneity, subgroup analysis and meta-regression was conducted. As summarized in Table 3, no significant statistically difference was found in subgroup analysis by covariates of continent (the study was from Asia, Europe, America, Africa, Oceania), detection method of iron (ICP-AES, AAS, other), and sample type (serum, plasma). However, a higher blood iron level was found in studies assessing iron levels with subjects in fasting status [SMD (95% CI): 0.208 (0.019, 0.397)], but not in studies with subjects in non-fasting status [SMD (95% CI): −0.346 (-0.907, 0.216)]. Univariate meta-regression suggested no covariate was responsible for between-study heterogeneity. *P* values of covariates of continent, food fasting, sample type, and tested methods of iron level were 0.68, 0.09, 0.14, and 0.78, respectively.

**TABLE 3:** Subgroup analysis of iron level in blood and Parkinson’s disease.

**Subgroup**	**Numbers of studies**	**SMD (95% CI)**	**Test of SMD = 0**		**Heterogeneity**	
			**Z**	***P* for Z**	***I*^2^ (%)**	***P* for *I*^2^**
**Continent**						
Asia	11	0.019(−0.362,0.399)	0.10	0.924	0.00%	0.000^∗∗^
Europe	7	−0.052(−0.446,0.342)	0.26	0.797	84.00%	0.000^∗∗^
**Food fasting**						
Yes	10	0.208(0.019,0.397)	2.16	0.031	67.00%	0.001^∗^
No	8	−0.346(−0.907,0.216)	1.21	0.227	96.40%	0.000^∗∗^
**Detection methods of iron**						
ICP-AES	7	−0.103(−0.826,0.620)	0.28	0.780	96.30%	0.000^∗∗^
AAS	4	0.042(−0.255,0.339)	0.28	0.782	78.00%	0.003^∗^
Other	7	−0.018(−0.237,0.201)	0.16	0.873	66.90%	0.006^∗^
**Sample**						
Serum	16	−0.124(−0.403,0.154)	0.88	0.381	90.1%	0.000^∗∗^
Plasma	2	0.477(−0.129,1.082)	1.54	0.123	94.0%	0.001^∗^

*SMD, standardized mean difference; CI, confidence interval; ^∗∗^*P* < 0.001, ^∗^*P* < 0.01 were considered significant.*

Egger’s test (*p* = 0.23) showed that no evidence of publication bias was found. In influence analyses, there was no significant change in the 95% CI after excluding any of the studies. This means no individual study had an excessive influence on the pooled effect. In cumulative meta-analysis showed in Figure 2B, blood levels of iron did not differ significant between PD patients and controls after including one paper in 1997. In sensitivity analysis, the pooled SMD were −0.14 (95% CI: −0.55–0.26, *I*^2^ = 85.5%, *P* = 0.000) for studies that excluded subjects with hepatopathy, nephropathy, atypical dietary habits, intake of iron supplements and chelating agents, as well as −0.02 (95% CI: −0.28–0.24, *I*^2^ = 55%, *P* = 0.084) for studies that excluded subjects with blood transfusions experience, blood donation anemia.

#### Iron Concentration Tends to Be Lower in CSF in Parkinson’s Disease

**FIGURE 3:**
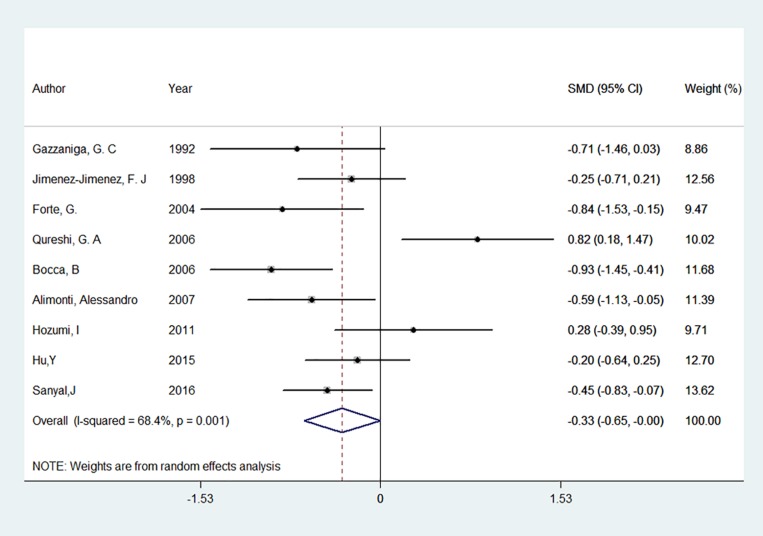
Forest plot of the Parkinson’s disease risk for cerebrospinal fluid level of iron. The standardized mean difference (SMD) with 95% confidence interval (CI) was calculated by using the random effects model.

Nine studies on CSF levels of iron between patients with PD and controls were involved in this meta-analysis (Gazzaniga et al., 1992; Logroscino et al., 1997; Jimenez-Jimenez et al., 1998; Forte et al., 2004; Bocca et al., 2006; Qureshi et al., 2006; Alimonti et al., 2007a; Hozumi et al., 2011; Sanyal et al., 2016), as well as 347 PD patients and 237 controls were involved. Among them, 2 study found an increase of iron levels (Logroscino et al., 1997; Hozumi et al., 2011), 2 found a decrease (Alimonti et al., 2007a; Sanyal et al., 2016), and 5 found no difference between PD patients and controls (Gazzaniga et al., 1992; Jimenez-Jimenez et al., 1998; Forte et al., 2004; Bocca et al., 2006; Qureshi et al., 2006). The pooled result showed that CSF levels of iron tend to be lower in PD patients compared with that in the controls (Figure 3). The pooled SMD from random effects model was −0.33 [95% CI:(−0.65, 0.00), *I*^2^ = 68.4%, *P* for *I*^2^ = 0.001]. Univariate meta-regression showed no covariate having impact on between-study heterogeneity, *p* values of covariates continent, food fasting, iron test methods were 0.53, 0.07 and 0.31 respectively. Egger’s test (*p* = 0.710) and visual inspection of the Egger’s funnel plot showed no evidence of small-study effects for all included studies.

## Discussion

The alteration of iron homeostasis in PD has been debated for decades. It has been accepted that iron accumulation in the substantia nigra in PD patients, but the alteration of iron levels in blood and CSF is not fully recognized. In the case-control study conducted by us, no significant difference was found in serum levels of iron between patients and controls. In the meta-analysis, no significant difference was found in blood levels of iron between patients and controls, and the cumulative meta-analysis found no difference after including one paper in 1997. The polled SMD did not change substantially in subgroup analyzes and sensitivity analyzes, except that a higher blood iron level was found in studies with subjects in fasting status. Meanwhile, iron levels in CSF tend to be lower in PD patients than in the controls both in the case-control study and the meta-analysis.

Abnormal deposition of iron in PD brains was first reported in 1924 (Lhermitte et al., 1924). The underlying mechanism for iron deposits in the substantia nigra remains unknown. It is still in debated that the alteration of cerebral iron homeostasis is the cause or the consequence of PD. Several epidemiological studies revealed that occupational exposure to iron or high intake of iron increased the risk for PD (Rybicki et al., 1993; Gorell et al., 1999; Powers et al., 2003). Prospective study showed that dietary non-heme iron intake from food was associated with a 30% increased risk for PD (Logroscino et al., 2008). A low intake of cholesterol in combination with high iron intake increased the risk of developing PD (Powers et al., 2009). Experiment conducted in mouse found that iron-overloaded models developed degeneration of dopaminergic neurons, and iron chelation prevented degeneration of midbrain dopaminergic neurons (Kaur et al., 2003, 2007; Devos et al., 2014). In clinical trials, iron chelation therapy for PD has shown significant improvement (Wang et al., 2017). All above evidence supports the hypothesis that iron is involved in the etiology of PD. However, several studies support the role of brain iron accumulation might be independent on the environmental exposure in the development of PD (Rivera-Mancia et al., 2010). Some studies failed to demonstrated the risk of iron exposure for PD (Webb et al., 2006), even showed that higher intake of iron might be protective against PD (Miyake et al., 2011). Non-significant difference in iron content has been found in brain tissues showing moderate neurodegeneration (Riederer et al., 1989), thus, it also could be considered that iron accumulation in PD might be the consequence of neuronal death since otherwise its levels should be increased since the early stages of the disorder. In this meta-analysis, we identified blood levels of iron did not differ significant between PD patients and controls. To some extend, this is in accordance with the hypothesis that brain iron accumulation may be independent on the environmental iron exposure in the development of PD.

In the present study, systemic serum iron metabolism was also measured. Lower ferritin level was observed in PD patients than controls, but no statistical significance was found in serum iron, TIBC, and transferrin saturation between them. Some studies reported that there is no association between iron metabolism and PD, for example, blood donations, which can decrease systemic iron stores, do not lower the risk of PD (Logroscino et al., 2006). However, a study of 213 PD patients and 219 healthy controls reported that ferritin, TIBC, and serum iron decreased gradually with the severity of the disease (Deng et al., 2017). The reasons for this variability in iron metabolism in PD patients may because that PD was a highly heterogeneous disease. This heterogeneity can manifest in many different ways, such as sex-specific differences, different genetic backgrounds, the course of disease, disease phenotype. For example, it has been reported that plasma transferrin level correlates with the tremor-dominant phenotype of Parkinson’s disease (Si et al., 2018), as well as the abnormally low iron in PD patients was especially pronounced for subjects of haptoglobin Hp 2-1 phenotype, which also have higher risk of PD (Costa-Mallen et al., 2015).

Both in the case-control study and the meta-analysis, iron levels in CSF tend to be lower in PD patients than in the controls. We hypothesized that iron deposition in PD patients in the basal ganglia, especially in the substantia nigra (SN), might lead to the decline of CSF iron levels. Several evidence have showed that elevated iron levels are key events leading to dopaminergic neuron degeneration in PD (Ke and Ming Qian, 2003; Kaur and Andersen, 2004; Ward et al., 2014). Increased iron levels were first observed in neuromelanin (NM)-containing neurons of the substantia nigra pars compacta (SNpc) in patients with PD, indicating that an iron-melanin interaction contributed to dopaminergic neurodegeneration in PD (Dexter et al., 1989; Bastian et al., 2010). Although the underlying mechanisms of iron accumulation in the SN are not fully clarified, it seemed resulted from a combination of increased iron import and decreased iron export. It was well known that two pathways were responsible for cellular iron uptake, the transferring (Tf)-transferrin receptor (TfR) pathway, and the non-transferrin-bound iron (NTBI) transport pathway. Tf-TfR is considered the major pathway, as well as the main NTBI pathway was divalent metal transporter 1 (DMT1), which was responsible for ferrous iron uptake. Several other metal transport systems such as the ferritin receptor, lactoferrin/lactoferrin receptor, and melanotransferrin are also involved in the NTBI pathway (Lopez et al., 2006; Konofal et al., 2007; Mackenzie et al., 2007). Ferroportin is the only known iron transporter responsible for cellular iron export to date (Ganz, 2005), which was first found to transport Fe^2+^ across the basolateral membrane of enterocytes with the auxiliary ferroxidase activity of ceruloplasmin or hephaestin (McKie et al., 2000; Le and Richardson, 2002). Amyloid precursor protein (APP) was shown to accelerate iron export from dopaminergic neurons by stabilizing ferroportin. In the substantia nigra of PD models, down-regulated of iron export protein Fpn1 and up-regulated of iron import protein DMT1 and TfR1 were observed, which played critical role in the iron accumulation in the substantia nigra (Kalivendi et al., 2003; Wang et al., 2007; Jiang et al., 2010). Neuromelanin also played an important role in the pathogenesis of Parkinson’s disease with high binding capacity for iron. The formation of reactive oxygen species through the Fenton reaction catalyzed by neuromelanin-bound iron ions subsequently lead to death of the dopaminergic cells in the substantia nigra (Knorle, 2018).

A main advantage of this study is abundant participants involved in the meta-analysis, making a more possibility to get reliable conclusions. Second, the pooled effects on all studies were consistent with most of the subgroup analysis and sensitive analysis. Third, little evidence of publication bias was detected in this meta-analysis, which indicated that our results were not affected by small-study effects. The limitations in this study should also be mentioned. First, high between-study heterogeneity was detected in the meta-analysis. The stratified analyses and meta-regression analysis did not find any of the following covariate, continents, fasting, sample type, and tested methods of iron as the important contributor to the between-study heterogeneity. Although before-mentioned covariates were not found to be the source of between-study heterogeneity, other possible related covariate, such as iron supplement usage, PD stage, PD complication, testing protocol of sample, etc., could not be excluded. Second, subgroup analysis was not performed on CSF iron levels because studies on CSF were not abundant. Third, the collection, storage and treatment condition of sample were diverse in different studies, which hint us to interpret the results with caution. Finally, the insufficient information provided by the included studies precluded the possibility of sensitive analysis in terms of disease status, phenotype, duration, and PD stage.

In summary, our study indicated blood levels of iron did not differ significant between PD patients and controls, but CSF levels of iron tended to be lower in PD patients compared with that in the controls. Further prospective studies are desired to clarify whether a causal link was existed between iron and PD.

## Data Availability

All datasets generated for this study are included in the manuscript and/or the supplementary files.

## Ethics Statement

The study protocol was approved by the Institutional Ethics Committee of Medical College of Qingdao University (2016N012), and conducted in accordance with the Declaration of Helsinki. All patients gave written informed consent to participate.

## Author Contributions

HJ and DZ designed the study. XS and HY performed the case-control study. XS performed the meta analysis. XS drafted the manuscript. HJ and DZ revised the manuscript. All authors approved the final version of the article, including the authorship list.

## Conflict of Interest Statement

The authors declare that the research was conducted in the absence of any commercial or financial relationships that could be construed as a potential conflict of interest.

## References

[B1] Ahmed S. S. Santosh W. (2010). Metallomic profiling and linkage map analysis of early Parkinson’s disease: a new insight to aluminum marker for the possible diagnosis. *PLoS One* 5:e11252. 10.1371/journal.pone.0011252 20582167 PMC2889819

[B2] Alimonti A. Bocca B. Pino A. Ruggieri F. Forte G. Sancesario G. (2007a). Elemental profile of cerebrospinal fluid in patients with Parkinson’s disease. *J. Trace Elem. Med. Biol.* 21 234–241. 10.1016/j.jtemb.2007.05.001 17980814

[B3] Alimonti A. Ristori G. Giubilei F. Stazi M. A. Pino A. Visconti A. (2007b). Serum chemical elements and oxidative status in Alzheimer’s disease, Parkinson disease and multiple sclerosis. *Neurotoxicology* 28 450–456. 10.1016/j.neuro.2006.12.001 17267042

[B4] Ayton S. Lei P. (2014). Nigral iron elevation is an invariable feature of Parkinson’s disease and is a sufficient cause of neurodegeneration. *Biomed. Res. Int.* 2014:581256. 10.1155/2014/581256 24527451 PMC3914334

[B5] Bastian T. W. Prohaska J. R. Georgieff M. K. Anderson G. W. (2010). Perinatal iron and copper deficiencies alter neonatal rat circulating and brain thyroid hormone concentrations. *Endocrinology* 151 4055–4065. 10.1210/en.2010-2252 20573724 PMC2940517

[B6] Bocca B. Alimonti A. Senofonte O. Pino A. Violante N. Petrucci F. (2006). Metal changes in CSF and peripheral compartments of parkinsonian patients. *J. Neurol. Sci.* 248 23–30. 10.1016/j.jns.2006.05.007 16765382

[B7] Bruggemann N. Hagenah J. Stanley K. Klein C. Wang C. Raymond D. (2011). Substantia nigra hyperechogenicity with LRRK2 G2019S mutations. *Mov. Disord.* 26 885–888. 10.1002/mds.23644 21312285 PMC3082617

[B8] Cabrera-Valdivia F. Jimenez-Jimenez F. J. Molina J. A. Fernandez-Calle P. Vazquez A. Canizares-Liebana F. (1994). Peripheral iron metabolism in patients with Parkinson’s disease. *J. Neurol. Sci.* 125 82–86. 7964893 10.1016/0022-510x(94)90246-1

[B9] Costa-Mallen P. Zabetian C. P. Agarwal P. Hu S. C. Yearout D. Samii A. (2015). Haptoglobin phenotype modifies serum iron levels and the effect of smoking on Parkinson disease risk. *Parkinsonism Relat. Disord.* 21 1087–1092. 10.1016/j.parkreldis.2015.07.006 26228081 PMC4554997

[B10] Deng Q. Zhou X. Chen J. Pan M. Gao H. Zhou J. (2017). Lower hemoglobin levels in patients with parkinson’s disease are associated with disease severity and iron metabolism. *Brain Res.* 1655 145–151. 10.1016/j.brainres.2016.11.007 27840188

[B11] Devos D. Moreau C. Devedjian J. C. Kluza J. Petrault M. Laloux C. (2014). Targeting chelatable iron as a therapeutic modality in Parkinson’s disease. *Antioxid. Redox Signal.* 21 195–210. 10.1089/ars.2013.5593 24251381 PMC4060813

[B12] Dexter D. T. Jenner P. Schapira A. H. Marsden C. D. (1992). Alterations in levels of iron, ferritin, and other trace metals in neurodegenerative diseases affecting the basal ganglia. The royal kings and queens parkinson’s disease research group. *Ann. Neurol.* 32 (Suppl.), S94–S100. 1510387 10.1002/ana.410320716

[B13] Dexter D. T. Wells F. R. Lees A. J. Agid F. Agid Y. Jenner P. (1989). Increased nigral iron content and alterations in other metal ions occurring in brain in Parkinson’s disease. *J. Neurochem.* 52 1830–1836. 10.1111/j.1471-4159.1989.tb07264.x 2723638

[B14] Dick F. D. De Palma G. Ahmadi A. Osborne A. Scott N. W. Prescott G. J. (2007). Gene-environment interactions in parkinsonism and Parkinson’s disease: the geoparkinson study. *Occup. Environ. Med.* 64 673–680. 10.1136/oem.2006.032078 17449559 PMC2078383

[B15] Drayer B. P. Olanow W. Burger P. Johnson G. A. Herfkens R. Riederer S. (1986). Parkinson plus syndrome: diagnosis using high field MR imaging of brain iron. *Radiology* 159 493–498. 10.1148/radiology.159.2.3961182 3961182

[B16] Farhoudi M. Taheraghdam A. Farid G. A. Talebi M. Pashapou A. Majidi J. (2012). Serum iron and ferritin level in idiopathic Parkinson. *Pak. J. Biol. Sci.* 15 1094–1097. 10.3923/pjbs.2012.1094.1097 24261127

[B17] Farlow J. Pankratz N.D. Wojcieszek J. Foroud T. (1993). “Parkinson disease overview,” in *GeneReviews* eds Pagon R. A. Adam M. P. Ardinger H. H. Wallace S. E. Amemiya A. Bean L. J. H. (Seattle, WA: University of Washington).

[B18] Forte G. Alimonti A. Pino A. Stanzione P. Brescianini S. Brusa L. (2005). Metals and oxidative stress in patients with Parkinson’s disease. *Ann. Ist. Super. Sanita* 41 189–195. 16244392

[B19] Forte G. Bocca B. Senofonte O. Petrucci F. Brusa L. Stanzione P. (2004). Trace and major elements in whole blood, serum, cerebrospinal fluid and urine of patients with Parkinson’s disease. *J. Neural Transm.* 111 1031–1040. 10.1007/s00702-004-0124-120 15254791

[B20] Fukushima T. Tan X. Luo Y. Kanda H. (2010). Relationship between blood levels of heavy metals and Parkinson’s disease in China. *Neuroepidemiology* 34 18–24. 10.1159/000255462 19893325

[B21] Fukushima T. Tan X. Luo Y. Wang P. Song J. Kanda H. (2013). Heavy metals in blood and urine and its relation to depressive symptoms in Parkinson’s disease patients. *Fukushima J. Med. Sci.* 59 76–80. 10.5387/fms.59.76 24500382

[B22] Ganz T. (2005). Cellular iron: ferroportin is the only way out. *Cell Metab.* 1 155–157. 10.1016/j.cmet.2005.02.005 16054057

[B23] Gazzaniga G. C. Ferraro B. Camerlingo M. Casto L. Viscardi M. Mamoli A. (1992). A case control study of CSF copper, iron and manganese in Parkinson disease. *Ital. J. Neurol. Sci.* 13 239–243. 10.1007/bf02224396 1624280

[B24] Gellein K. Syversen T. Steinnes E. Nilsen T. I. Dahl O. P. Mitrovic S. (2008). Trace elements in serum from patients with Parkinson’s disease–a prospective case-control study: the nord-trondelag health study (HUNT). *Brain Res.* 1219 111–115. 10.1016/j.brainres.2008.05.002 18538747

[B25] Gerlach M. Ben-Shachar D. Riederer P. Youdim M. B. (1994). Altered brain metabolism of iron as a cause of neurodegenerative diseases? *J. Neurochem.* 63 793–807. 10.1046/j.1471-4159.1994.63030793.x 7519659

[B26] Good P. F. Olanow C. W. Perl D. P. (1992). Neuromelanin-containing neurons of the substantia nigra accumulate iron and aluminum in Parkinson’s disease: a LAMMA study. *Brain Res.* 593 343–346. 10.1016/0006-8993(92)91334-b 1450944

[B27] Gorell J. M. Johnson C. C. Rybicki B. A. Peterson E. L. Kortsha G. X. Brown G. G. (1999). Occupational exposure to manganese, copper, lead, iron, mercury and zinc and the risk of Parkinson’s disease. *Neurotoxicology* 20 239–247. 10385887

[B28] Gorell J. M. Peterson E. L. Rybicki B. A. Johnson C. C. (2004). Multiple risk factors for Parkinson’s disease. *J. Neurol. Sci.* 217 169–174. 14706220 10.1016/j.jns.2003.09.014

[B29] Hagemeier J. Geurts J. J. Zivadinov R. (2012). Brain iron accumulation in aging and neurodegenerative disorders. *Expert Rev. Neurother.* 12 1467–1480. 10.1586/ern.12.128 23237353

[B30] Hagenah J. M. Becker B. Bruggemann N. Djarmati A. Lohmann K. Sprenger A. (2008). Transcranial sonography findings in a large family with homozygous and heterozygous PINK1 mutations. *J. Neurol. Neurosurg. Psychiatry* 79 1071–1074. 10.1136/jnnp.2007.142174 18469032

[B31] Hagenah J. M. Konig I. R. Becker B. Hilker R. Kasten M. Hedrich K. (2007). Substantia nigra hyperechogenicity correlates with clinical status and number of Parkin mutated alleles. *J. Neurol.* 254 1407–1413. 10.1007/s00415-007-0567-y 17934880

[B32] Hegde M. L. Shanmugavelu P. Vengamma B. Rao T. S. S. Menon R. B. Rao R. V. (2004). Serum trace element levels and the complexity of inter-element relations in patients with Parkinson’s disease. *J. Trace Elem. Med. Biol.* 18 163–171. 10.1016/j.jtemb.2004.09.003 15646263

[B33] Higgins J. P. Thompson S. G. Deeks J. J. Altman D. G. (2003). Measuring inconsistency in meta-analyses. *BMJ* 327 557–560. 10.1136/bmj.327.7414.557 12958120 PMC192859

[B34] Hozumi I. Hasegawa T. Honda A. Ozawa K. Hayashi Y. Hashimoto K. (2011). Patterns of levels of biological metals in CSF differ among neurodegenerative diseases. *J. Neurol. Sci.* 303 95–99. 10.1016/j.jns.2011.01.003 21292280

[B35] Hu Y. Yu S. Y. Zuo L. J. Piao Y. S. Cao C. J. Wang F. (2015). Investigation on abnormal iron metabolism and related inflammation in Parkinson disease patients with probable RBD. *PLoS One* 10:e0138997. 10.1371/journal.pone.0138997 26431210 PMC4592206

[B36] Jiang H. Song N. Xu H. Zhang S. Wang J. Xie J. (2010). Up-regulation of divalent metal transporter 1 in 6-hydroxydopamine intoxication is IRE/IRP dependent. *Cell Res.* 20 345–356 10.1038/cr.2010.20 20125122

[B37] Jimenez-Jimenez F. J. Molina J. A. Aguilar M. V. Meseguer I. Mateos-Vega C. J. Gonzalez-Munoz M. J. (1998). Cerebrospinal fluid levels of transition metals in patients with Parkinson’s disease. *J. Neural Transm.* 105 497–505. 9720977 10.1007/s007020050073

[B38] Kalivendi S. V. Kotamraju S. Cunningham S. Shang T. Hillard C. J. Kalyanaraman B. (2003). 1-Methyl-4-phenylpyridinium (MPP+)-induced apoptosis and mitochondrial oxidant generation: role of transferrin-receptor-dependent iron and hydrogen peroxide. *Biochem. J.* 371(Pt 1), 151–164. 10.1042/BJ20021525 12523938 PMC1223270

[B39] Kaur D. Andersen J. (2004). Does cellular iron dysregulation play a causative role in Parkinson’s disease? *Ageing Res. Rev.* 3 327–343. 10.1016/j.arr.2004.01.003 15231240

[B40] Kaur D. Peng J. Chinta S. J. Rajagopalan S. Di Monte D. A. Cherny R. A. (2007). Increased murine neonatal iron intake results in Parkinson-like neurodegeneration with age. *Neurobiol. Aging* 28 907–913. 10.1016/j.neurobiolaging.2006.04.003 16765489

[B41] Kaur D. Yantiri F. Rajagopalan S. Kumar J. Mo J. Q. Boonplueang R. (2003). Genetic or pharmacological iron chelation prevents MPTP-induced neurotoxicity in vivo: a novel therapy for Parkinson’s disease. *Neuron* 37 899–909. 10.1016/s0896-6273(03)00126-0 12670420

[B42] Ke Y. Ming Qian Z. (2003). Iron misregulation in the brain: a primary cause of neurodegenerative disorders. *Lancet Neurol.* 2 246–253. 10.1016/s1474-4422(03)00353-3 12849213

[B43] Knorle R. (2018). Neuromelanin in Parkinson’s Disease: from fenton reaction to calcium signaling. *Neurotox. Res.* 33 515–522. 10.1007/s12640-017-9804-z 28879408

[B44] Konofal E. Cortese S. Marchand M. Mouren M. C. Arnulf I. Lecendreux M. (2007). Impact of restless legs syndrome and iron deficiency on attention-deficit/hyperactivity disorder in children. *Sleep Med.* 8 711–715. 10.1016/j.sleep.2007.04.022 17644481

[B45] Kumudini N. Uma A. Devi Y. P. Naushad S. M. Mridula R. Borgohain R. (2014). Association of Parkinson’s disease with altered serum levels of lead and transition metals among South Indian subjects. *Indian J. Biochem. Biophys.* 51 121–126. 24980015

[B46] Le N. T. Richardson D. R. (2002). Ferroportin1: a new iron export molecule? *Int. J. Biochem. Cell Biol.* 34 103–108. 10.1016/s1357-2725(01)00104-2 11809412

[B47] Lee D. W. Andersen J. K. (2010). Iron elevations in the aging Parkinsonian brain: a consequence of impaired iron homeostasis? *J. Neurochem.* 112 332–339. 10.1111/j.1471-4159.2009.06470.x 20085612

[B48] Lhermitte J. Kraus W. M. McAlpine D. (1924). on the occurrence of abnormal deposits of iron in the brain in parkinsonism with special reference to its localisation. *J. Neurol. Psychopathol.* 5 195–208. 10.1136/jnnp.s1-5.19.195 21611545 PMC1068263

[B49] Li W. Langkammer C. Chou Y. H. Petrovic K. Schmidt R. Song A. W. (2015). Association between increased magnetic susceptibility of deep gray matter nuclei and decreased motor function in healthy adults. *Neuroimage* 105 45–52. 10.1016/j.neuroimage.2014.10.009 25315786 PMC4339282

[B50] Lill C. M. Klein C. (2017). Epidemiology and causes of Parkinson’s disease. *Nervenarzt* 88 345–355. 10.1007/s00115-017-0288-280 28289797

[B51] Logroscino G. Chen H. Wing A. Ascherio A. (2006). Blood donations, iron stores, and risk of Parkinson’s disease. *Mov. Disord.* 21 835–838. 10.1002/mds.20826 16453313

[B52] Logroscino G. Gao X. Chen H. Wing A. Ascherio A. (2008). Dietary iron intake and risk of Parkinson’s disease. *Am. J. Epidemiol.* 168 1381–1388. 10.1093/aje/kwn273 18945687 PMC2727188

[B53] Logroscino G. Marder K. Graziano J. Freyer G. Slavkovich V. LoIacono N. (1997). Altered systemic iron metabolism in Parkinson’s disease. *Neurology* 49 714–717. 10.1212/wnl.49.3.714 9305329

[B54] Lopez V. Suzuki Y. A. Lonnerdal B. (2006). Ontogenic changes in lactoferrin receptor and DMT1 in mouse small intestine: implications for iron absorption during early life. *Biochem. Cell Biol.* 84 337–344. 10.1139/o06-059 16936804

[B55] Mackenzie B. Takanaga H. Hubert N. Rolfs A. Hediger M. A. (2007). Functional properties of multiple isoforms of human divalent metal-ion transporter 1 (DMT1). *Biochem. J.* 403 59–69. 10.1042/BJ20061290 17109629 PMC1828886

[B56] Madenci G. Bilen S. Arli B. Saka M. Ak F. (2012). Serum iron, vitamin B12 and folic acid levels in Parkinson’s disease. *Neurochem. Res.* 37 1436–1441. 10.1007/s11064-012-0729-x 22367474

[B57] Mariani S. Ventriglia M. Simonelli I. Bucossi S. Siotto M. Donno S. (2016). Association between sex, systemic iron variation and probability of Parkinson’s disease. *Int. J. Neurosci.* 126 354–360. 10.3109/00207454.2015.1020113 26000822

[B58] McKie A. T. Marciani P. Rolfs A. Brennan K. Wehr K. Barrow D. (2000). A novel duodenal iron-regulated transporter, IREG1, implicated in the basolateral transfer of iron to the circulation. *Mol. Cell.* 5 299–309. 10.1016/s1097-2765(00)80425-6 10882071

[B59] Miyake Y. Tanaka K. Fukushima W. Sasaki S. Kiyohara C. Tsuboi Y. (2011). Dietary intake of metals and risk of Parkinson’s disease: a case-control study in Japan. *J. Neurol. Sci.* 306 98–102. 10.1016/j.jns.2011.03.035 21497832

[B60] Mochizuki H. Yasuda T. (2012). Iron accumulation in Parkinson’s disease. *J. Neural Transm.* 119 1511–1514. 23070727 10.1007/s00702-012-0905-9

[B61] Nischwitz V. Berthele A. Michalke B. (2008). Speciation analysis of selected metals and determination of their total contents in paired serum and cerebrospinal fluid samples: an approach to investigate the permeability of the human blood-cerebrospinal fluid-barrier. *Anal. Chim. Acta* 627 258–269. 10.1016/j.aca.2008.08.018 18809082

[B62] No author list (2013). Higher iron concentrations may protect against Parkinson’s disease. *BMJ* 346:f3691. 10.1136/bmj.f3691 23761187

[B63] Pichler I. Del Greco F. Goegele M. Lill C.M. Bertram L. Do C.B. (2013). Serum iron levels and the risk of Parkinson disease: a mendelian randomization study. *PLoS Med.* 10:e1001462. 10.1371/journal.pmed.1001462 23750121 PMC3672214

[B64] Powers K. M. Smith-Weller T. Franklin G. M. Longstreth W. T. Jr. Swanson P. D. (2003). Parkinson’s disease risks associated with dietary iron, manganese, and other nutrient intakes. *Neurology* 60 1761–1766. 10.1212/01.wnl.0000068021.13945.7f 12796527

[B65] Powers K. M. Smith-Weller T. Franklin G. M. Longstreth W. T. Jr. Swanson P. D. (2009). Dietary fats, cholesterol and iron as risk factors for Parkinson’s disease. *Parkinsonism Relat. Disord.* 15 47–52. 10.1016/j.parkreldis.2008.03.002 18424169 PMC2751763

[B66] Qureshi G. A. Qureshi A. A. Memon S. A. Parvez S. H. (2006). Impact of selenium, iron, copper and zinc in on/off Parkinson’s patients on L-dopa therapy. *J. Neural Transm. Suppl.* 2006 229–236. 10.1007/978-3-211-33328-0_24 17447433

[B67] Riederer P. Sofic E. Rausch W. D. Schmidt B. Reynolds G. P. Jellinger K. (1989). Transition metals, ferritin, glutathione, and ascorbic acid in parkinsonian brains. *J. Neurochem.* 52 515–520. 10.1111/j.1471-4159.1989.tb09150.x 2911028

[B68] Rivera-Mancia S. Perez-Neri I. Rios C. Tristan-Lopez L. Rivera-Espinosa L. Montes S. (2010). The transition metals copper and iron in neurodegenerative diseases. *Chem. Biol. Interact.* 186 184–199. 10.1016/j.cbi.2010.04.010 20399203

[B69] Rybicki B. A. Johnson C. C. Uman J. Gorell J. M. (1993). Parkinson’s disease mortality and the industrial use of heavy metals in michigan. *Mov. Disord.* 8 87–92. 10.1002/mds.870080116 8419812

[B70] Sanyal J. Ahmed S.S. Ng H.K. Naiya T. Ghosh E. Banerjee T.K. (2016). Metallomic biomarkers in Cerebrospinal fluid and Serum in patients with Parkinson’s disease in Indian population *Sci. Rep.* 6:35097. 10.1038/srep35097 PMC5067653 27752066

[B71] Schweitzer K. J. Brussel T. Leitner P. Kruger R. Bauer P. Woitalla D. (2007). Transcranial ultrasound in different monogenetic subtypes of Parkinson’s disease. *J. Neurol.* 254 613–616. 10.1007/s00415-006-0369-367 17415511

[B72] Si Q. Q. Yuan Y. S. Zhi Y. Tong Q. Zhang L. Zhang K. (2018). Plasma transferrin level correlates with the tremor-dominant phenotype of Parkinson’s disease. *Neurosci. Lett.* 684 42–46. 10.1016/j.neulet.2018.07.004 29981876

[B73] Sian-Hulsmann J. Mandel S. Youdim M. B. Riederer P. (2011). The relevance of iron in the pathogenesis of Parkinson’s disease. *J. Neurochem.* 118 939–957. 10.1111/j.1471-4159.2010.07132.x 21138437

[B74] Sofic E. Paulus W. Jellinger K. Riederer P. Youdim M. B. (1991). Selective increase of iron in substantia nigra zona compacta of parkinsonian brains. *J. Neurochem.* 56 978–982. 10.1111/j.1471-4159.1991.tb02017.x 1704426

[B75] Sofic E. Riederer P. Heinsen H. Beckmann H. Reynolds G. P. Hebenstreit G. (1988). Increased iron (III) and total iron content in post mortem substantia nigra of parkinsonian brain. *J. Neural Transm.* 74 199–205. 10.1007/bf01244786 3210014

[B76] Speziali M. Di Casa M. (2009). Copper, iron, zinc and other element concentrations in cerebrospinal fluid of Parkinson’s disease patients - considerations on literature data. *Trace Elem. Electrolytes* 26 171–176. 10.5414/tep26171

[B77] Squitti R. Gorgone G. Panetta V. Lucchini R. Bucossi S. Albini E. (2009). Implications of metal exposure and liver function in parkinsonian patients resident in the vicinities of ferroalloy plants. *J. Neural Transm.* 116 1281–1287. 10.1007/s00702-009-0283-280 19680597

[B78] Torsdottir G. Kristinsson J. Sveinbjornsdottir S. Snaedal J. Johannesson T. (1999). Copper, ceruloplasmin, superoxide dismutase and iron parameters in Parkinson’s disease. *Pharmacol. Toxicol.* 85 239–243. 10.1111/j.1600-0773.1999.tb02015.x 10608487

[B79] Wang C. Fan G. Xu K. Wang S. (2013). Quantitative assessment of iron deposition in the midbrain using 3D-enhanced T2 star weighted angiography (ESWAN): a preliminary cross-sectional study of 20 Parkinson’s disease patients. *Magn. Reson. Imaging* 31 1068–1073. 10.1016/j.mri.2013.04.015 23746648

[B80] Wang J. Jiang H. Xie J. X. (2007). Ferroportin1 and hephaestin are involved in the nigral iron accumulation of 6-OHDA-lesioned rats. *Eur. J. Neurosci.* 25 2766–2772. 10.1111/j.1460-9568.2007.05515.x 17561842

[B81] Wang N. Jin X. Guo D. Tong G. Zhu X. (2017). Iron chelation nanoparticles with delayed saturation as an effective therapy for parkinson disease. *Biomacromolecules* 18 461–474. 10.1021/acs.biomac.6b01547 27989126

[B82] Ward R. J. Zucca F. A. Duyn J. H. Crichton R. R. Zecca L. (2014). The role of iron in brain ageing and neurodegenerative disorders. *Lancet Neurol.* 13 1045–1060. 10.1016/S1474-4422(14)70117-70116 25231526 PMC5672917

[B83] Webb T. Whittington J. Holland A. J. Soni S. Boer H. Clarke D. (2006). CD36 expression and its relationship with obesity in blood cells from people with and without prader-willi syndrome. *Clin. Genet.* 69 26–32. 10.1111/j.1399-0004.2006.00536.x 16451133

[B84] Youdim M. B. Riederer P. (1993). The role of iron in senescence of dopaminergic neurons in Parkinson’s disease. *J. Neural Transm. Suppl.* 40 57–67. 8294901

[B85] Yu X. Du T. Song N. He Q. Shen Y. Jiang H. (2013). Decreased iron levels in the temporal cortex in postmortem human brains with Parkinson disease. *Neurology* 80 492–495. 10.1212/WNL.0b013e31827f0ebb 23303856 PMC3590051

[B86] Zhao H. W. Lin J. Wang X. B. Cheng X. Wang J. Y. Hu B. L. (2013). Assessing plasma levels of selenium, copper, iron and zinc in patients of Parkinson’s disease. *PLoS One* 8:e83060. 10.1371/journal.pone.0083060 24340079 PMC3858355

